# Some Notes on the Etiology of Pyorrhœa Alveolaris

**Published:** 1894-10

**Authors:** Junius E. Cravens

**Affiliations:** Indianapolis, Ind.


ARTICLE II.

### SOME NOTES ON THE ETIOLOGY OF PYORRHŒA ALVEOLARIS.

BY JUNIUS E. CRAVENS, D. D. S., INDIANAPOLIS, IND.

Since last January there have been published original communications on this subject, in various dental journals, from the pens of no less than fifteen distinguished members of our profession. When one considers that no two of the fifteen distinguished offered similar opinions except upon a few abstract points, one naturally feels at sea, and a blanked big, deep sea at that. Therefore, as the journals for several months have been so badly afflicted with Rigg's disease, I may perhaps be excused for having caught the infection to the extent of writing a book upon this disease, and telling dentists how to cure it, a point that was omitted by the fifteen. It seemed to me to be the one point most needed.

The etiology of a disease treats of its cause; therefore the cause of phorrhœa alveolaris or Rigg's disease has been selected for my subject in a desire to bring up the rear of the procession of fifteen who have preceded me upon this text. The manner of cure of this disease is clearly given in the book which I have written.

Pyorrhœa alveolaris involves both pericementum and and periosteum, but each is affected in a manner peculiar.

The immediate origin of the disease is in an inflammation of the periosteum intimately surrounding the orifice of the socket and on the surface of the ridge of alveolar process (orbicular periostitis). If the periostitis remains external to the socket, there will be a case of *gingivitis*—nothing more and nothing serious. But, should the periostitis penetrate the socket, as it usually will in time, if unchecked, there will evenuate a case of pyorrhœa alveolaris (Rigg's disease), which always is something serious.

The causes ef gingival periostitis are many, but the principal one is encroachment of salivary calculus under the margin of the gum, a thing that cannot occur unless the gum is *inconstant.* Inconstancy of gum may be due to any of many things and conditions. The individual may be physically run down, weak, flaccid; he may have recently recovered from a prolonged attack of typhoid fever, or pneumonia, or inflammatory rheumatism, etc.

Inconstancy of gum may be due to mechanical effects such as too vigorous and careless digging about the teeth with wooden picks; injuries to the margin of the gum by stiff brushes; by deep and often unnecessary adjustment of rubber dam, sustained for hours by heavy ligatures or steel clamps, bruising and crowding away the gum; the alternating pressure of ill-fitting partial dentures. Any sustained injury of the orbicular gum margin may result in permanent looseness of the gingiva and thus invite an attack of pyorrhœa alveolaris.

Inconstancy of gum, if general in a mouth, would indicate a constitutional obliquity extending far back of any observed local conditions. A constitutional obliquity (a diathesis) may be hereditary or acquired; an acquired obliquity may be temporary, usually is; a hereditary obliquity may be corrected, but seldom is.

Then, to begin with, we have a constitutional obliquity that is manifested in the mouth by inconstancy of gum about the teeth—it is not well attached; saliva is admitted and naturally tartar forms a bit of crust there; the periosteum about the tooth becomes inflamed; the gum shows high congestion with swollen margin, a condition that is called *gingivitis.* Gingivitis passes over into the socket, and leads on to pyorrhœa alveolaris. *All of this begins with inconstancy of gum, from any cause.*

A constitutional obliquity that has been acquired may pass away under competent medical attention, but the pyorrhœa alveolaris that has resulted from its temporary existence will remain to plague and destroy.

Pyorrhœa alveolaris, once established, is self-sustaining, and its "pockets," although cured, are slow in filling up; they do not always fill up, but a healing around and to the roots does occur, and the scar tissue established is forever proof against a recurrence of the disease.

The pus "pockets" are formed before the perosteum of the socket and the pericementum of the root part company; they are forced to so fall apart, thus leaving an intervening gap to be filled by something; saliva flows in and contributes its mite of lime salts, vagrant scales of epithelium, and general oral rubbish; plasma weeps from the periosteum into the "pocket," pus corpuscles escape into it; there is pus, purulence, putrefaction, with more lime salts and organic products deposited from the pus—constituting *pyonal calculus.*

The fluid contents within the "pocket" induce further resorption of the alveolar process and consequent enlargement of the pus cavity; then follows more pus, more pyonal calculus, until at last the road divides; one branch leads to cure, the other to the forceps. I do not believe there ever was a case of hereditary pyorrhœa alveolaris.—*Western Dental Journal.*

